# Innate Immune Response to Tick-Borne Pathogens: Cellular and Molecular Mechanisms Induced in the Hosts

**DOI:** 10.3390/ijms21155437

**Published:** 2020-07-30

**Authors:** Alessandra Torina, Sara Villari, Valeria Blanda, Stefano Vullo, Marco Pio La Manna, Mojtaba Shekarkar Azgomi, Diana Di Liberto, José de la Fuente, Guido Sireci

**Affiliations:** 1 Istituto Zooprofilattico Sperimentale della Sicilia, Via Gino Marinuzzi 3, 90100 Palermo, Italy; alessandra.torina@izssicilia.it (A.T.); sara.villari@izssicilia.it (S.V.); stefano.vullo@izssicilia.it (S.V.); 2 Central Laboratory of Advanced Diagnostic and Biological Research (CLADIBIOR), BIND, University Hospital “Paolo Giaccone”, Università degli studi di Palermo, Via del Vespro 129, 90100 Palermo, Italy; marcopio.lamanna@unipa.it (M.P.L.M.); mojtaba.shekarkarazgomi@unipa.it (M.S.A.); diana.diliberto@unipa.it (D.D.L.); guido.sireci@unipa.it (G.S.); 3 SaBio, Instituto de Investigación en Recursos Cinegéticos IREC-CSIC-UCLM-JCCM, Ronda de Toledo s/n, 13005 Ciudad Real, Spain; JosedeJesus.Fuente@uclm.es; 4 Department of Veterinary Pathobiology, Center for Veterinary Health Sciences, Oklahoma State University, Stillwater, OK 74078, USA

**Keywords:** inflammasome, innate immune response, tick borne pathogens, gene ontology analysis

## Abstract

Many pathogens are transmitted by tick bites, including *Anaplasma* spp., *Ehrlichia* spp., *Rickettsia* spp., *Babesia* and *Theileria* sensu stricto species. These pathogens cause infectious diseases both in animals and humans. Different types of immune effector mechanisms could be induced in hosts by these microorganisms, triggered either directly by pathogen-derived antigens or indirectly by molecules released by host cells binding to these antigens. The components of innate immunity, such as natural killer cells, complement proteins, macrophages, dendritic cells and tumor necrosis factor alpha, cause a rapid and intense protection for the acute phase of infectious diseases. Moreover, the onset of a pro-inflammatory state occurs upon the activation of the inflammasome, a protein scaffold with a key-role in host defense mechanism, regulating the action of caspase-1 and the maturation of interleukin-1β and IL-18 into bioactive molecules. During the infection caused by different microbial agents, very similar profiles of the human innate immune response are observed including secretion of IL-1α, IL-8, and IFN-α, and suppression of superoxide dismutase, IL-1Ra and IL-17A release. Innate immunity is activated immediately after the infection and inflammasome-mediated changes in the pro-inflammatory cytokines at systemic and intracellular levels can be detected as early as on days 2–5 after tick bite. The ongoing research field of “inflammasome biology” focuses on the interactions among molecules and cells of innate immune response that could be responsible for triggering a protective adaptive immunity. The knowledge of the innate immunity mechanisms, as well as the new targets of investigation arising by bioinformatics analysis, could lead to the development of new methods of emergency diagnosis and prevention of tick-borne infections.

## 1. Introduction

Tick-borne diseases are emerging infectious diseases caused by bacterial, viral and parasitic pathogens, including the bacteria *Anaplasma* spp., *Ehrlichia* spp. and *Rickettsia* spp., and the protozoa *Babesia* spp. and *Theileria* spp. [1].

*Anaplasma* species (order Rickettsiales, family Anaplasmataceae) are obligate intracellular pathogens, which survive in host cells avoiding the host immune response [2]. The genus includes species affecting companion, domestic and wildlife animals. *Anaplasma marginale* and *Anaplasma phagocytophilum* are two relevant pathogenic species, with the latter also able to infect humans [3]. Other species pathogenic towards animals are *Anaplasma bovis*, *Anaplasma centrale*, *Anaplasma ovis* and *Anaplasma platys* [4].

*Ehrlichia* species (order Rickettsiales, family Anaplasmataceae) are able to infect several vertebrate hosts [5]. *Ehrlichia* genus includes six species: *E. chaffeensis*, *E. ewingii*, *E. canis*, *E. muris*, *E. ruminantium* and *E.ovis* that affect several vertebrate hosts [6,7,8]. *E. chaffeensis* and *E. ewingii* are also important zoonotic agents [9,10]. *Ehrlichia* efficiently establishes an intracellular infection and avoids immune defenses in vertebrate and invertebrate hosts through complex molecular and cellular reprogramming strategies [11,12].

*Rickettsia* genus (order Rickettsiales, family Rickettsiaceae) includes obligate intracellular bacteria causing increasingly emerging human febrile diseases, including Mediterranean Spotted Fever, Rocky Mountain Spotted Fever, Epidemic typhus, murine typhus, scrub typhus [13]. Within the *Rickettsia* genus, Spotted Fever Group comprises two main pathogens: *R. rickettsii*, responsible for Rocky Mountain Spotted Fever, the most severe rickettsioses in the western hemisphere, and *R. conorii*, responsible for Mediterranean Spotted Fever, whose spreading and severity is increasing throughout Europe and Africa [14]. The Typhus Group includes the pathogenic agents of epidemic typhus (*R. prowazekii*) and murine typhus (*R. typhi*), displaying severe outbreaks worldwide. After a tick bite, bacteria replication within the infection site can lead to a necrotic lesion (eschar) and, subsequently, endothelial cells are the first cellular targets for rickettsia infection, with vascular severe damages.

*Babesia* sensu stricto (Order Piroplasmida, family Babesiidae) includes infecting agents of a wide range of domestic and wild animals [15,16]. It comprehends both the so-called large (*B*. *caballi*, *B*. *bigemina*, *B*. *canis*, *B*. *rossi*, *B*. *vogeli*) and small (*B*. *gibsoni*, *B*. *bovis*) *Babesia* species [17]. The pathogens infect red blood cells where they reproduce asexually.

*Theileria* sensu stricto species (order Piroplasmida, family Theileridae) are the aetiologic agents of a variety of diseases in domestic and wild ruminants [16,18]. They include all the *Theileria* species pathogens for cattle, i.e. *T. annulata*, *T. parva*, *T. orientalis* [17]. After the tick bite, injected sporozoites infect leukocytes and multiply inside them by merogony. Mature schizonts develop into merozoites, which are released and invade erythrocytes, forming piroplasms [19].

The interaction of tick-transmitted pathogens with the host immune system has been widely studied [20,21,22,23,24,25].

In the present review, we discuss the main interactions of the above-mentioned pathogens with different effector mechanisms of the host innate immunity, with a particular attention to the activation of the inflammasome, the leading actor of innate immunity. The review also discusses new targets of investigation arising by bioinformatics analysis.

## 2. Inflammasomes as Key Multimolecular Mechanisms Reacting to Infections

The innate immune signalling structures, the so called innate immune sensors, include Toll-like receptors (TLRs), Nod-like receptors (NLR), absent in myeloma (AIM2), C-type lectin receptors, retinoid acid-inducible gene I-like receptors (RIG I-like) and cyclic GMP-AMP synthase (cGAS)/STING (stimulator of interferon genes) [26,27]. The best analyzed pathways are those activated from TLR and NLR receptors, which localize and respond to antigens either on membrane surface or inside the cells, respectively [28]. TLRs are pattern recognition receptors, which sense a broad range of microbial ligands leading to expression of genes involved in inflammation and other immune responses [29], while NLR proteins are cytosolic pathogen recognition receptors (PRRs) able to oligomerize into a large inflammasome. Inflammasome is a protein scaffold with a key-role in host defence mechanism regulating the action of caspase-1 (CASP1) and the maturation of interleukin-1β (IL-1β) and IL-18.

The inflammasome activation was shown to be induced during infections by Gram negative- (i.e., *Escherichia coli*), Gram positive- (i.e., *Staphylococcus aureus*), Gram variable-bacteria (i.e., *Mycobacterium tuberculosis*), DNA- (i.e., *Adenovirus*), RNA-virus (i.e., *Hepatitis C virus*), Fungi (i.e., *Aspergillus Fumigatus*) and Protozoa (*Leishmania* spp.) [30,31].

**Figure 1:**
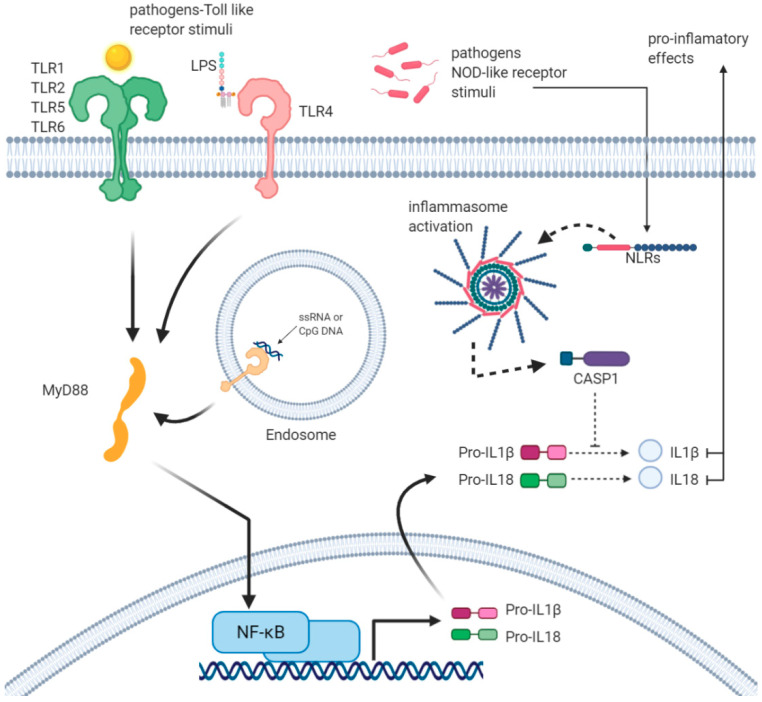
Inflammasomes activation and production of IL1β and IL18: Surface-expressed TLRs, such as TLR1, 2, 5, and 6, bind TLR-dependent stimuli, for example bacterial PAMPs. Following this binding, TLRs, through the adaptor protein MyD88, activate the transcription factor NF-κB to induce the expression of inflammatory genes, such as IL1β and IL18, leading to the production of pro-IL1β and pro-IL18. On the other hand, different PAMPs or DAMPs activate the inflammasome through various NLRs, such as NLRP3. Inflammasome leads to active Caspase-1 that processes pro-IL1β/pro-IL18, leading to the active form of IL1β and IL18. Active cytokines leave the cell and act as pro-inflammatory molecules.

Upon activation, multimeric complexes assemble to function as activation platforms for the autoproteolysis of CASP1, a protease which cleaves pro- IL-1β and IL-18 into their mature forms [32] (Figure 1).

The most well-established inflammasomes are NLRP1 (nucleotide-binding domain leucine-rich repeat-containing [NLR] family, pyrin domain [PYD]-containing 1), NLRP3 (NLR family, PYD-containing 3), NLRC4 (NLR family, caspase activation and recruitment domain [CARD]-containing 4), AIM2 (absent in melanoma 2), and pyrin. It was reported that other NLR family proteins, including NLRP6 and NLRP9b, may also form functional inflammasomes [33].

In particular, TLR2 promotes NLRP3 inflammasome activation providing a priming signal (signal 1) necessary for activation of the inflammasome by a second potassium-depleting signal (signal 2) [29].

Activation of inflammasomes causes a sequence of responses, including release of IL-1β andIL-18 and the induction of pyroptotic, or inflammatory, cell death through cleavage of gasdermin D (GSDMD) [34,35]. GSDMD is a pore-forming protein, which cleavage by the inflammatory caspases critically determines pyroptosis by releasing the cleaved gasdermin-N domain that bears intrinsic pyroptosis-inducing activity [36]. In particular, active CASP1 enzymatically cleave GSDMD into two fragments (the N domain and C domain). The GSDMD-N domain can form pores on lipid membranes, drive K+ efflux and induce pyroptosis through cell membrane disruption [37,38].

Inflammasomes can interact directly with the inflammatory effector CASP1 through CARDs or by utilizing the adaptor apoptosis-associated speck-like (ASC) protein to mediate the interaction between PYD-containing sensors and CARD-containing CASP1.

Non-canonical inflammasome activation has two main effects: (1) the induction of pyroptosis and (2) the secretion of the pro-inflammatory cytokines IL-1β and IL-18 via the activation of the canonical NLRP3 [39,40,41]. GSDMD is the effector molecule linking these two downstream processes [34,35]. Indeed, murine CASP11 (CASP4 and CASP5 in humans) oligomerizes upon binding with cytosolic LPS and becomes active. Active CASP4/5 cleaves GSDMD to drive pyroptosis and NLRP3 inflammasome-dependent cleavage of CASP1 [42]. CASP4-mediated NLRP3 activation depends on potassium efflux [43].

Some recent studies showed that LPS is not directly recognized by caspases but through a receptor called guanylate-binding protein 1 (GBP-1), which protects in a cell-autonomous manner against infection with bacteria, parasites and viruses and promotes the activation of human CASP4 upon transfection of LPS [44]. Moreover, GBP1 acts as a cytosolic LPS sensor detecting and targeting the LPS-containing membranes of Gram-negative bacteria, where it assembles a platform that promotes CASP4 recruitment and activation [45].

An oversimplification of the interactions between canonical and non canonical inflammasomes could happen as follows: the canonical inflammasomes, through the activation of CASP1, generate the mature forms of IL1β and IL18 while the non canonical inflammasomes, activated by LPS, through bioactivity of CASP4 contributes with IL1β and IL18 to inflammatory processes and pyroptosis.

There are probably aspects of inflammasome biology, which may be elucidated in the context of infections with uncommon pathogens. Ticks transmitted pathogens are deeply different from other, more commonly studied microbes, especially for their life strategy and induced pathology. For example, many of them induce a less severe disease compared to the pathogens commonly activating inflammasome. Some tick-borne bacteria do not display canonical PAMPs, such as lipopolysaccharides (LPS) [46,47,48].

## 3. Innate Immune Response to Tick-Borne Pathogens as the First and, in Many Cases, Resolutive Mechanisms of Protection

*Anaplasma* genus show no evidence of peptidoglycan or LPS [49], molecules typically identified by the innate immune system through recognition receptors expressed by macrophages or neutrophils, in order to clear the pathogens. More precisely, peptidoglycan and LPS have been eliminated at the genomic level through reductive evolution, as they lost genes for their biosynthesis [50]. Consequently, their absence could allow *Anaplasma* to infect the host without activating any response, thus persisting in its cells without triggering an effective innate immune response. Nevertheless, persistent infections cannot be identified in cases with low bacteremia.

*A. phagocytophilum* binds to sialylated scaffold proteins on neutrophil and granulocyte surface, adhering to polymorphonuclear leukocytes (PMNs) via P-selectin glycoprotein ligand 1 (PSGL-1), which is used to engage P-selectin displayed on inflamed endothelium during rolling adhesion. After bacterial internalization, the endosome ceases to mature and *A*. *phagocytophilum* divides until cell lysis or discharge [51]. Infection results in significant disruption or inhibition of normal neutrophil functions, including endothelial cell adhesion, motility, apoptosis, IFN-γ signaling pathways, respiratory burst and phagocytosis. In vivo responses are dominated by IFN-γ and IL-10, while they lack tumor necrosis factor α (TNFα) and IL-4. IFN-γ activates macrophages production (nitric oxide, reactive oxygen species, TNFα, phagocytosis) causing cytotoxic effects [49]. As a result, infection in humans and in animal models leads to a macrophage activation syndrome (MAS) [52] where a key role in severity of tissue damage is related to high serum levels of IL-10, IL-12, ferritin and especially IFN-γ [20,31,49,50], secreted by NK and NKT cells, but also by a component of adaptive immunity, CD8 T lymphocytes.

As for *A. marginale,* vascular endothelial cells could serve as reservoirs for the bacteria and pass them on to PMNs, which are recruited to the feeding lesion by pro-inflammatory cytokines, without any returning to the circulatory system. In fact, *A. marginale* could easily infect red blood cells in the microcapillary environment, where there is intimate contact between erythrocytes and endothelial cells. Thus, a reservoir of *Anaplasma* in endothelium may provide an opportunity for the ongoing, direct cell-to-cell infection of blood cells, and at the same time avoid host immune effectors such as antibodies and complement [53].

Major surface proteins (MSPs) are crucial for the interactions between *A. marginale* and host cells, especially adhesins, which mediate binding and entry into host cells, are essential for survival. *A. marginale* mostly uses its invasins OmpA, Asp14, and AipA to infect cells [54]. *A. marginale* passes into host cells by endocytosis and it exhibits a biphasic developmental cycle. After entrance, the infectious dense form evolves into the non-infectious reticulated form, thus originating ample rickettsiae colonies by binary fission. The non-infectious form then reconverts into the dense form, which is released from cells and survives extracellularly, thus invading naive host cells and thereby initiating new infections.

NK cells actively contribute to the host defence against *Ehrlichia* when primary infection occurs. Wildtype C57BL/6 mice, that already overcame primary infection with *Ehrlichia muris*, showed a better protection versus *Ixodes ovatus Ehrlichia* (IOE), a highly virulent *Ehrlichia* strain causing fatal disease in naïve mice. Habib and colleagues [22] showed that this protective memory against IOE was abolished if NK cells were depleted in such *E. muris*-primed mice, leading to 80% of mice deceased to the infection, similar to naïve mice infected with the same dose of IOE. NK cell-depleted mice showed also a smaller number of *Ehrlichia*-specific IFN-γ-producing memory CD4^+^ and CD8^+^ T-cells and a low titre of *Ehrlichia*- specific antibodies, suggesting a role for NK even in the adaptive response activation. In the same study, recipient Rag2^−^/^−^Il2rg^−^/^−^mice, lacking T, B and NK cells, which were transferred with NK cells from *E. muris*-primed mice, showed a higher survival rate suggesting that NK cells from *E. muris*-primed mice may acquire memory-like phenotype.

As concerning *Ehrlichia* interaction with macrophages, Zhang and collaborators [55] showed that *E. chaffeensis* is able to significantly modify gene expression in a THP1 human monocyte cell line. For example, *E. chaffeensis* is able to inhibit the transcription of cytokines as IL-12, IL-15, and IL-18 acting in the early innate immune response. These cytokines are involved in stimulating TH1 and NK cells to produce IFN-γ, which activates macrophages [56]. This downregulation could be useful to allow *Ehrlichia* survival against macrophages. Other mechanisms induced by *E. chaffeensis* included the up-regulation of Nuclear factor-kB (NF-kB) and apoptosis inhibitors, cyclins and CDK, to promote host cell survival and inhibition of proteins involved in membrane trafficking. A different mechanism recently discovered involves an *Ehrlichia*-dependent induction of a strong pro-inflammatory response leading to the cleavage of CASP1 in bone marrow–derived dendritic cells (BMDCs) and bone marrow derived–macrophages (BMDMs), promoting IL-1b, IL-1a and type I interferon (IFN-I) production. IFN-I increases host susceptibility to fatal ehrlichiosis, favouring *Ehrlichia*-induced immunopathology and replication. Possible mechanisms include inflammasome activation, autophagy reduction, suppression of protective CD4^+^ T cells and NKT-cell responses against the pathogen [57].

IFN-γ is a relevant macrophage-activating cytokine with a role in inhibiting *E. chaffeensis* infection at early stages. This cytokine is able to decrease iron availability, necessary for intracellular bacteria survival [11]. However, IFN-γ has an effective anti-ehrlichial role only in the early stages of the infection, while it is ineffective when the infection is established. This result is due to the *Ehrlichia*-induced expression of transferrin receptor on the host cell surface and disruption of the IFN-γ induced signalling that involves the Janus kinase (JAK) and the signal transducer and activator of transcription (STAT) pathways. In particular, in the inhibition of IFN-γ induced tyrosine phosphorylation of STAT1, JAK1, and JAK2 a role for Tandem repeat protein 47 (TRP47) was reported though the interaction with the Protein Tyrosine Phosphatase, Non-Receptor Type 2 (PTPN2) [58]. Tandem repeat proteins are secreted serine/threonine-rich proteins showing antibody epitopes in the tandem repeat regions [59,60,61]. TRPs interact with a diverse network of host proteins involved in many host cellular processes including cell signalling, transcriptional and translational regulation, post-translational modification, intracellular trafficking, cytoskeletal organization, and apoptosis [11]. Through the interaction with several host proteins, TRPs contribute to the ehrlichial evasion from the host immune response and to the infection establishment [62]. In particular, TRP47 is an ehrlichial effector protein that interacts with multiple host cell proteins essential for cellular entry and survival.

As mentioned above, the first event in *Rickettsia* infection is the entry and replication within ECs [63], an infectious process including adhesion, endocytosis, endosome escape, replication in cytosol and release of bacteria (via focal lysis for SFG *Rickettsia*, via cell lysis for TG *Rickettsia* and via budding for *O. tsustugamushi*). *Rickettsia* in ECs leads to the production of a plethora of inflammatory mediators. As an example, ECs release CCL5/RANTES, CCL2/MCP-1 and CXCL8/IL-8, three chemokines responsible for chemo-attraction of leucocytes, especially monocytes and neutrophils [64]. Furthermore, the upregulation of VCAM1, ICAM-1, αVβ3 integrin, E-selectin in ECs supports transmigration and adhesion of leucocytes. Regarding the recruitment of NK cells and T cells, ECs produce both CXCL10/IP10 and CXCL9/MIG capable of recruiting T lymphocytes and NK cells. Since CXCL9 and CXCL10 have been demonstrated to act via CXCR3 [65], and CXCR3 has been suggested to be present in NKT cells, the further recruitment and action of NKT cells in local *Rickettsia*-related inflammatory event may be suggested [66]. Following chemo-attraction of innate and adaptive immune cells, ECs are further responsible for a promotion of inflammation via the production and release of relevant cytokines, as TNFα, IL-1β and IL-6, leading to further triggering of both innate and adaptive immune cells activity. Of importance, further inflammatory mediators secreted by ECs include prostaglandins, leading to classical alteration of vascular permeability and edema following rickettsial infections [67], an event potentiated by endothelial denudation of vessels, COX-2 production and nitric oxide (NO) production by damaged endothelium.

Regarding NK cells, they have been widely studied in the context of rickettsial infection. Undoubtedly, NK cells are closely involved in early innate immunity against *Rickettsia*, and different studies have discovered a peculiar role for NK cells in IFN-γ secretion, prior to the same release by late activation of T cells [68]. IFN-γ is of clear relevance in early immune defense against *Rickettsia*, acting on ECs and macrophages for promotion of bacterial killing via nitrergic pathways, tryptophan depletion, pathogen growth inhibition and other mechanisms [69,70,71]. IFN-γ has a wide range of effects including enhancement of NK cells activity, augmented expression of MHC-I and MHC-II on Antigen-Presenting Cells (APCs), activation of inducible NOS, promotion of phagocytic activity [67]. Enhanced NK cells activity and their increased IFN-γ production have been demonstrated for SFG rickettsiae, especially in *R. conorii*, with a main role of NK-derive IFN-γ-mediated immunity compared to cytotoxicity [72]. Regarding TG *Rickettsia*, although enhanced NK cell activity has also been reported during early rickettsia infection, the contribution of NK cells to a relevant production of IFN-γ has been questioned by the observation of unaltered IFN-γ release after NK cell depletion in mice, suggesting that other cell sources may sustain IFN-γ production (i.e. tissue macrophages) [73]. In contrast, infection by *O. tsustugamushi* has been showed to be correlated to an increase in NK cells; both CD69^+^ and CD69^−^ NK cells were found in patient blood samples, but, importantly, NK CD69^+^ cells produce a larger amount of IFN-γ than CD69^−^ ones [74]. In a study by Keller and colleagues [75] an increase of serum IFN-γ in a murine experimental model has been revealed, and in vitro studies demonstrated the role of IFN-γ in bacterial killing via induction of iNOS in macrophages. In summary, the NK cells-IFN-γ pathway is of importance in rickettsial infection, although further studies in humans are needed, especially for TG rickettsiae.

NKT cells are an emerging group of immune cells bridging innate and adaptive immunity, displaying both killing and cytokine-producing properties [76]. Although the presence of NKT-activating ligands as α-linked glycuronylceramide and glycosyldiacylglycerol have been demonstrated in *Rickettsia* [77], the contribution of such subset of immune cells in rickettsial infection is still overlooked. In a study involving an experimental mouse model for *R. conorii* infection [78], an increased number of NKT cells have been reported in the spleen of infected mice at 3 days post-infection. Of relevance, a significant increase of IL-17^+^ NKT cells were reported compared to a not relevant percentage of IFN-γ^+^ NKT cells. Thus, cytokine microenvironment in *R. conorii* infection may favour the development of IL-17^+^ cells, possibly sustaining the onset of immune response, although the protective versus pathogenic role of such subset will require further in-depth investigations.

As previously discussed, apart from endothelial cells, rickettsiae may invade other target cells especially tissue macrophages (MΦ). *R. akari* has been reported to promote the MΦ production of IL-6, TNF-α and IL-1β, as well as to induce upregulation of nuclear factor kappa-light-chain-enhancer of activated B cells (NF-kB) [79]. Pathogen recognition receptors as TLR2 and TLR4 have been identified as signal transduction receptors for *R. akari*. In particular, TLR4 seems to be essential for *Rickettsia* recognition given that its absence in KO mice leads to fatal disease at non-lethal doses [80]. Experimental studies showed that *R. australis* infection results in MΦ release of a plethora of cytokines including IL-1β, IL-6, TNF-α, IL-12p40, IL-18, which are reduced in MyD88 KO mice, lacking TLR-related MyD88 signaling pathway [81]. Importantly, bactericidal activity by macrophages have been attributed both to cytokine release and to NO production. However, apart from different studies confirming the capability of MΦ to effectively mediate rickettsia clearance, a hypothesis on the exploitation of MΦ by some *Rickettsia* species for dissemination throughout the host tissues has been also proposed [74]. In addition, MΦ activity has been addressed to contribute deeply to pathogenic overactivation of immune system, suggesting that immune homeostasis in rickettsial infection represents a major issue for the host organism [82].

Regarding DC, in an interesting study by Fang et al. [83], the differential role of bone-marrow derived DCs (BDMCs) have been revealed in mice resistant (B6) or susceptible (C3H) to *R. conorii* infection. Importantly, BMDC from B6 mice were more potent in internalizing and processing *Rickettsia*, leading to a proficient IL-12p40 production and to priming of CD4^+^ T cells. Instead, BDMCs from C3H mice displayed a late CD4^+^ T-cell activation with development of immunosuppressive Foxp3^+^ T Regulatory cells (T Regs), whose activation may explain the major infection susceptibility of C3H mice compared to B6. Such observations confirmed the necessity of a subtle regulation of the immune system during rickettsial infection, whose disturbance may influence the shift from a protective immune response to a pathogenic immune response by overwhelming immunity [84]. In addition, further studies on KO mice have showed that TLR4-mediated recognition of *Rickettsia* by DCs could represent a fundamental event in initiating and promoting the early immune response [71], suggesting that the definition of the exact role of TLRs in rickettsial infection should be addressed to depict a complete picture on early immunity related to rickettsiae.

Antigens derived from microbes could promote immune response due to the activation of effector mechanisms of innate immune response such as complement, natural killer cells, phagocytosis, receptors for molecular profiles. In this section, different types of interactions between tick-transmitted microbes and innate immunity will be discussed. Innate immune mechanisms could promote a rapid type of protection that could neutralize tissue damages induced by pathogens during the acute phase of infectious diseases.

Recognition of microbial compounds by host immune system could be due to receptors for molecular profiles such as TLRs mainly produced on APCs. It was reported that a lipid fraction of *B. bovis*-infected erythrocytes is able to stimulate macrophage response in bovine [85]. Moreover, a different phlogistic response was induced by the lipids of the attenuated vaccine strain of *B. bovis* R1A (LA) and the pathogenic *B. bovis* strain S2P (LV) in murine peritoneal macrophages. LA and particularly its fractions of phosphatidic acid and phosphatidylserine plus phosphatidylinositol (PS + PI), induced an activation of these cells with lipid body formation, cyclooxygenase-2, TNF-α and IL-6 secretion. However, the comparison from wild type and TLR2 deficient (TLR2KO) mice demonstrate that TLR2 mediate macrophage activation by the lipid fractions [86].

Another branch of innate immunity is constituted by the complement proteins. *B. gibsoni* ribosomal phosphoriboprotein P0 (rBgP0) previously reported to be a cross-protective antigen against *Babesia* infection, was used to immunize C57BL/6 wild-type (WT) and C3-deficient mice. Following the immunization with rBgP0, WT mice induced a specifically strong humoral response consisting of mixed immunoglobulins IgG1 and IgG2 associated with high production of IFN-γ in the supernatant of splenocytes. While C3KO mice had significantly decreased total IgG, IgG1 and IgG2b responses, the secretions of IL-12 and IFN-γ tended to be lower than those in WT mice. Partial protection was only observed in rBgP0-immunized WT mice, but not in C3KO mice or controls. Indeed, rBgP0-immunized WT mice showed significant reductions in the initiation of parasitaemia correlated with delayed mortalities and considerable survival rates [87].

An additional interesting field of study of innate immunity interaction with microorganism antigens is the characterization of DC and NK and their interactions. It was shown that bovine NK cells can respond to microbial-exposed splenic DC with both regulatory and effector activities. The results demonstrated the influence of accessory DC phenotype and maturation state on the ability to induce NK cell activity along with the importance of the microbial agent being processed. In addition, NK cell activation by DC is cell-to-cell contact-dependent. These data are supported by immunohistological evidence for DC/NK cell contact during the early response to *B. bovis* infection [23]. Two subsets of DC were characterized in cattle. The phenotypic profile of each population was CD13^+^ and CD14^+^, respectively. The two cell populations differed in their ability to produce nitric oxide and had a different pattern of cytokine mRNA when stimulated with *Mycobacterium bovis* BCG or *Babesia bovis* [88].

A different lymphocyte subset involved in innate immunity is constituted by lymphocytes with TCR γδ. Regarding the distribution of lymphocytes in the spleen of *Babesia*-infected spleen, the role of these cells is still not clear, but bovine WC1^−^ γδT cells express CD2 and CD8. This subset can produce IFN-γ in response to cytokine stimulation, and it is found in largest proportion into spleen and intestine. The observed accumulation of these cells in the red pulp of naive calves infected with *B. bovis* is consistent with their expected role in the transition from innate to acquired immunity [89].

NK quick response could be useful to protect hosts in the acute phase of babesiosis. Innate immunity was reacting to antigens for 2 weeks after infection of calves with *B. bovis*. Each calf that survived from the acute disease responded by increasing systemic IFN-γ and type-1 cytokine, macrophage and splenic CD8^+^ activation. The activated CD8^+^ cells display a bioactivity of NK-like cells, and the expansion occurred with an increase in IL-15 mRNA in the spleen [90]. The specific splenic cell types producing IFN-γ in response to infection and the cellular factors regulating the induction have not been fully determined. Splenic NKp46^+^ cells were identified and purified. They consisted of CD3^−^, CD2^+/−^, and CD8^+/−^ populations. NK cells responded to exogenous IL-12 and IL-18 with the production of IFN-γ. Functionally, IL-18 served as a potent co-stimulant with IL-12 for IFN-γ production [24].

Moreover, a strong innate spleen-dependent immune response was observed in calves to infection with *Babesia* parasites and it was related to the protection of young calves from clinical consequences of the infection. For example, when a first *B. bovis* infection occurs in young cattle they usually have no clinical consequences and a long-lasting immunity is found. On the contrary, a severe illness is induced in older cattle [91]. As concerning this mechanism, it was reported for *B. bovis* that in young calves the infection with virulent strains of the pathogen is followed by an early production of IL-12 and IFN-γ, occurring before the influence of IL-10 and associated to protection. In the spleen of adult cattle, in contrast, delayed and lesser levels of IL-12 and IFN-γ mRNA expression were observed and they decreased following IL-10 expression. A CD8^+^ T-cell expansion was revealed in both calves and adults, while an antibody response was elicited only in calves [92].

Innate immunity is involved in the initial response against *T. annulata* infection. NK cells and several plasma proteins, including the proteins of the complement system, mediate the early defence against the pathogen. In addition, the innate immune system enhances adaptive immune responses towards the infectious agent. *T. annulata*-infected cells stimulate polyclonal naïve T lymphocyte proliferation by mechanisms involving NF-kB, MAPK and PI3-K-PKB pathways, followed by the up-regulation of pro-inflammatory cytokines and MHC class II molecules [93].

In particular, the pathway of NF-kB, one of the most important cellular factors involved in the regulation of the host innate antimicrobial response, is targeted directly by the schizont in both the transformant species *T. parva* and *T. annulata*. This factor is essential for protection against apoptosis and it contributes to maintaining a continuous proliferation, regulating the expression of anti-apoptotic proteins and downregulating tumor-suppressor genes. Its inhibition results in immediate apoptosis of *Theileria*-transformed cells [94]. *Theileria* parasites induce the constitutive NF-kB activation by the recruitment of large aggregates of IkB kinase (IKK) complexes to the surface of the transforming schizont. IKK is the regulator of NF-kB pathways and acts phosphorylating cytoplasmic inhibitors of NF-kB that are, subsequently, polyubiquitinated and degradated, allowing NF-kB to translocate to the nucleus [95].

Aberrantly activated T lymphocytes switch to a Th1 phenotype, producing large quantities of IFN-γ [96], which, together with the excessive production of pro-inflammatory cytokines, is mainly responsible for parasite-induced pathology [97]. In details, it was reported that *T. parva*-transformed cells produce exclusively IFN-γ, whereas *T. annulata*-infected cells express type I IFN, particularly IFN-β [98]. Other proinflammatory cytokines expressed by *T. annulata*-infected cells included IL-1α, IL-1β, IL-6, TNFα [99]. Following *T. annulata*-activation, macrophages elicit NO production, which can inhibit invasion and proliferation of the pathogen in host cells, even if, an excessive NO production plays a prominent role in the pathogenesis of the disease [100].

Moreover, several studies have reported a role in host immune responses against *T. annulata* infections for TLRs, an important group of pattern recognition receptors, whose activation leads to the induction of IFNs and cytokines acting in host defence and pathogenesis [101]. In particular, it was found that mRNA expression of TLR10 was significantly higher in *T. annulata*-infected PBMCs from the more resistant indigenous cattle (*Bos indicus*) with respect to crossbreds, suggesting a role for TLR10 in enhancing the host immune responses in *T. annulata* infections [102].

Recently, the transcriptional profiles of genes involved in the TLR and NLR signalling pathways in response to *T. annulata* infection were investigated at different sampling times during the infection course. Authors found significant differences in the transcription levels of TLR1, TLR6, TLR10, NLRP1, and MyD88 genes and their downstream signalling molecules in samples collected from 72 h to 168 h following *T. annulata* infection, compared to pre-infection values. The serum concentrations of IL-6, IL-1β, and TNFα significantly increased at 96 h and 168 h postinfection. These results may contribute in determining the mechanisms of TLR and NLR signalling pathways in immune response against *T. annulata* [103].

## 4. Gene Ontology Analysis of Interactions between Innate Immune Response and Tick-Borne Pathogens

**Figure 2:**
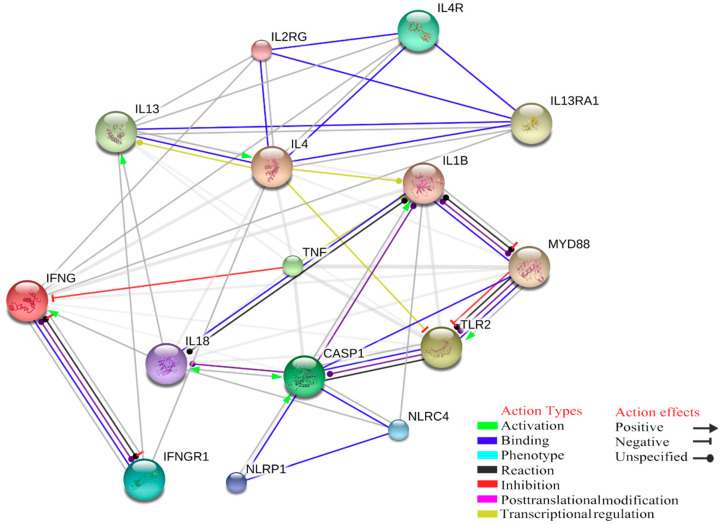
Interaction among IFN-γ, TLR2, CASP1, TNFα and IL-4 that may have effect on inflammasome assembly and activation during tick-borne pathogens infection.

Statistical overrepresentation analysis of enriched genes can reveal high significance in association with Gene Ontology (GO) terms for biological function, clearly reflecting a molecular signature of innate immune response to tick-borne pathogens, in particular the inflammasome activation. At least six NLRP Nucleotide-binding domain and Leucine rich Repeat-containing receptors (NLRP) with different activation stimuli functions, such as bacterial toxins able to active NLRP3 or lipopeptides activating stimuli on NLRP7, can trigger CASP1. Therefore, inflammasomes can be formed by several different NLR family sensors. As explained above, the tick-borne pathogen infections are not controlled by innate immunity, but a pro-inflammatory state occurs upon innate immune cells activation of the inflammasome via TLR2, leading to the mature forms of IL-1β and IL-18, cleaved by caspases, which consequently cause a macrophages activation. Based on protein-chemical interactions, the correlation between inflammasome and macrophages activation pathways is not direct, as they are mediated by apoptosis-related cysteine peptidase 1 (CASP1). Macrophage activation is controlled by IFN-γ signalling pathways and tick-borne pathogens infection leads to a MAS, caused by a significant increase of IFN-γ secretion induced by the transcription of cytokines like IL-12, IL-15, and IL-18 (Figure 2). The increasing of IFN-γ has a positive transcriptional regulation or activation effect on STAT1, which contributes to the transcriptional up-regulation of a number of genes involved in the inflammasome activation.

Furthermore, the TNFα and IL-4 play a critical role in this downregulation and in MAS. As can be seen in Figure 1, IL-4 has a negative transcriptional regulation action on toll-like receptor 2, while the expression of TNFα has inhibition action on IFN-γ; consequently, this balance may have effect on the inflammasome assembly. To better understand the role of this inhibition in human and animal infection models, we chose some biological terms to create an annotation data set to analyse the weak part of innate immunity, which cannot control the infection in its early onset. These Gene Ontology (GO) terms are: interferon-gamma secretion (GO:0072643), inflammasome complex (GO:0061702), transferrin receptor activity (GO:0004998), macrophage activation (GO:0042116), and toll-like receptor signaling pathway (GO:0002224). A total number of 2388 of annotations were recorded (Table 1).

Based on this gene set, the GO analysis was run on PANTHER GO-slim Biological Process on False Discovery Rate (FDR), with *p* < 0.05. Then, the GO biological process, which is related to innate immunity, was chosen with a total number of 166 genes observed (Table 2).

Another role in inducing the expression of Transferrin receptor and iron control is played by iron ion transport genes, with seven genes involved in our dataset (TCIRG1, TFR2, TFRC, ATP6V1G2, LTF, TF, SLC11A1). Moreover, in our gene interaction analysis, we showed that transferrin has a positive activation on IL-18.

As explained before, the increasing of IFN-γ can cause MAS, as NK cells display a pivotal role in early innate immunity against rickettsial infection by IFN-γ secretion via different pathways. We also chose some biological terms for creating an annotation dataset to study how NK cells and IFN-γ secretion lead to an early innate immune response against *Rickettsia* or other pathogens. These terms were: natural killer cell activation (GO:0030101), regulation of interferon-gamma secretion (GO:1902713), interferon-gamma secretion (GO:0072643), response to redox state (GO:0051775), tryptophan metabolic process (GO:0006568), nitric-oxide synthase activity (GO:0004517), regulation of interleukin-17 secretion (GO:1905076). A total of 202 annotations were recorded for these gene ontology terms (Table 3).

Based on this gene set, GO analysis was run on PANTHER GO-slim Biological Process on FDR with *p* < 0.05. Then GO biological process was chosen with a total number of 202 genes observed. Our GO study and gene product interaction showed that at the level of high confidence interaction score (0.700), there is an significant interaction score between Myeloid Differentiation Primary Response Protein (MyD88) and the TLR and IL-1 receptor signalling pathway in the innate immune response (Figure 1). Furthermore, the interaction between these two biological functions (IFN-γ production/regulation of NK-mediated immunity) probably represent the hotlink between macrophages and NK cells innate immune response. Based on experimental and biochemical data, posttranslational modifications between MyD88 and JAK2 were identified.

**Figure 3:**
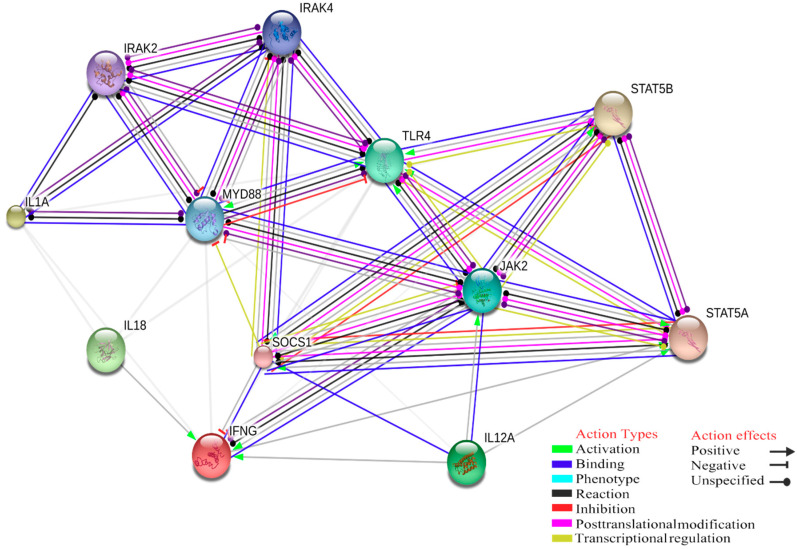
Interaction among MyD88, Toll-like receptor 4, IL-1 receptor signalling pathway in the innate immune response to tick-borne pathogens that could positively control the IFN-γ secretion; meanwhile, there is lack of IL-18 and IL-12 due to MAS.

In our dataset, there is a significant score for JAK2 receptor involved in various processes either in innate or adaptive immunity, consequently leading to IFN-γ production where there is a lack of IL-12A and IL-18 due to MAS, for example in response to *A. phagocytophilum* infection (Figure 3).

**Figure 4:**
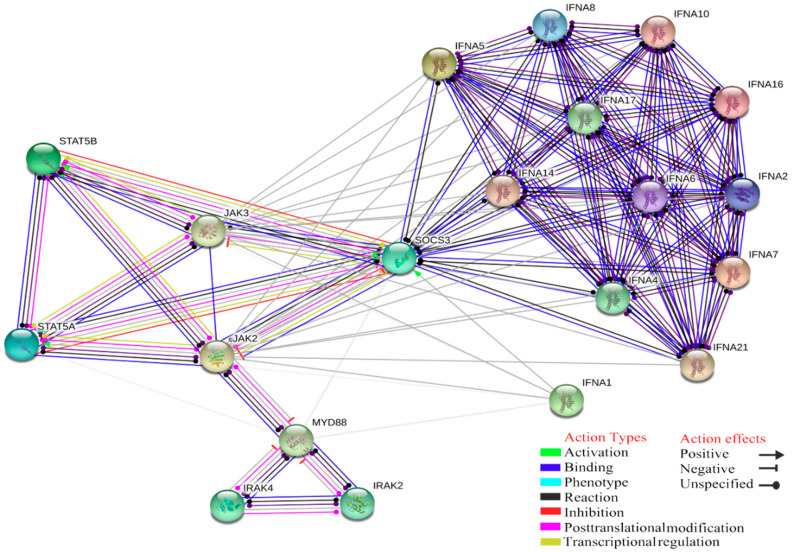
Interaction between MyD88 and JAK-STAT pathway, linked to IFN-α; this interaction is mediated by suppressors of cytokine signalling 3, SOCS family proteins which are part of a classical negative feedback system regulating cytokine signal transduction.

Another essential role of MyD88 and the JAK-STAT pathways is linked to IFN-α (IFNA1, IFNA4, IFNA5, IFNA6, IFNA7, IFNA8, IFNA10, IFNA14, IFNA16, IFNA17, IFNA20, IFNA21). These genes are linked with a biological function known as natural killer cell activation that is involved in immune response and positive regulation of IFN-γ due to NK activated by specific antigens or mitogens, displaying antiviral activity (GO:0002323, raw *p*-value = 7.23 × 10^−23^, FDR = 6.77 × 10^−21^; Figure 4).

**Figure 5:**
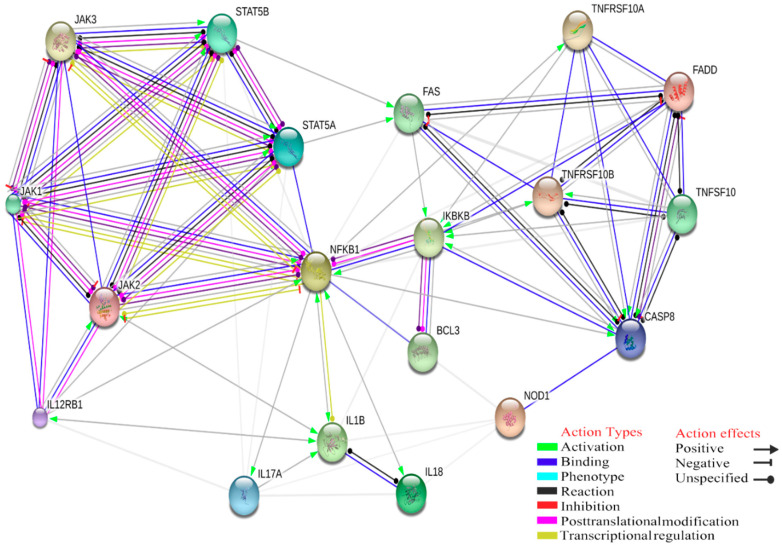
Interaction between IL-1β and I-kB kinase/NF-kB signalling pathway, which have positive activation of Nuclear Factor Kappa B Subunit 1. NF-kB is a pleiotropic transcription factor present in almost all cell types. It is the endpoint of a series of signal transduction events initiated by a vast array of stimuli related to many biological processes such as inflammation, immunity, differentiation, cell growth, tumorigenesis and apoptosis. Positive activation of CASP8 (score: 0.900).

Moreover, our gene interactions study showed that IL-12, STAT5A and STAT5B have a negative inhibition on IL-17 and positive activation on FAS (TNF receptor superfamily, member 6), which consequently leads to activation of CASP8, apoptosis-related cysteine peptidase (significant score: 0.567) (Figure 5).

As discussed above, one of the mechanisms affected by *E. chaffeensis* is the up-regulation of NF-kB1 and apoptosis inhibitors. Based on our dataset, the critical gene in this network is Nucleotide Binding Oligomerization Domain Containing 1 (NOD1), as there is evidence for post-translational modification and expression with inhibition effect on NF-kB1, although this interaction is not direct but mediated by receptor-interacting serine-threonine kinase 2 (RIPKI2). Even if our Gene Ontology computation is mainly analyzing human and mouse genome, it was found that the same models of inflammasome dysregulation and interactions with other molecules were detectable in different species [104]. Clearly, deeper molecular analysis in ruminants, dogs and cats are needed to assess how inflammasomes-side effects could be relevant in cells from several species.

## 5. Conclusions

Even though recent studies have shed light on the role of innate immunity during tick-borne infections, much remains unknown. Host immune response could interact with *Babesia* spp., *Rickettsia* spp., *Anaplasma* spp., *Ehrlichia* spp. and *Theileria* spp. using different cells and/or molecules responsible for the innate immune response onset. Effector mechanisms of innate immune responses, displayed by host in short time, are able to inhibit symptoms in the acute infection phases, but they do not contain the infection. Instead, this task is up to T and B cell responses, mounted by the immune system afterwards and able to induce a long-term protection against pathologies induced by tick-borne microbes.

Tick-transmitted microbes differ both physiologically and in their pathogenic potential from other well-characterized pathogens. This is likely the result of the exposition of TBPs to the evolutionary pressures of complex interactions among vertebrate hosts and vectors [105,106]. As previously proposed [107], tick-host-pathogen molecular interactions evolved as conflict and cooperation with mutual beneficial effects for all parts. These interactions evidence coevolutionary mechanisms by which pathogens manipulate tick vector and animal host protective responses to facilitate the infection and transmission of the disease.

An example of this selective pressure may be the ability of *A. phagocytophilum* to persist in immune-competent hosts between seasons of tick activity. This ability is the result of a complex and coordinated interaction that have led the bacterium to reduce its genomes to only essential genes allowing for nearly infinite numbers of recombined antigens and macromolecular exchange with its host cell [3,108,109]. Another finding supporting the close co-evolutionary relationship among microorganisms, ticks and animal hosts resulted by a recent geographically extensive phylogenetic study on groEL sequences of *A. phagocytophilum,* showing a considerable spread of some of its haplotypes and the affinity of some others towards well-defined groups of vertebrates, ticks and regions [110].

Despite some tick-borne pathogens lack of PAMPs, they may elicit inflammasome activation by inducing a dysregulated state within the host cell by causing aberrant compartmentalization of molecules, proteins and/or lipids. Consequently, the downstream inflammasome signalling culminates in proinflammatory cytokine secretion [111] and changes in their plasma levels can be detected as early as on days 2–5 after tick bite. The GO analysis underlined five key points that need to be deeply characterized to better understand the role of innate immunity in response to tick borne pathogens. These key points include IFN-γ secretion, inflammasome complex, transferrin receptor activity, macrophage activation and TLR signalling pathway. IFN-γ secretion, TLR signalling and macrophage activation in immune system of hosts infected by tick-borne pathogens were previously studied but a more profound analysis of these mechanisms needs to be performed for an optimal understanding of the role of these pathways in immunity against tick-borne pathogens. Future research projects need to better characterize the role of the transferrin receptor pathway in innate immune response to tick-borne pathogens.

We hypothesize that research in the fields of inflammasome as well as transferrin receptor pathway, will advance the discovery of mechanistic details of inflammasome activation and innate immunity, which may ultimately be related with disease progression and immunological resistance to tick-borne infections. The deep knowledge of the innate immunity mechanisms could lead to design new strategies of diagnosis and prevention of tick-borne diseases and allow the assessment of possible new immunotherapies for these infectious diseases.

## Figures and Tables

**Table 1:** Total number of GO Annotations for THE study of innate immune responses and tick-borne pathogens, which might be playing a critical role in TNF alpha and IL-4 downregulation and Macrophage Activation Syndrome.

GO ID	GO Terms	Number of Genes Associated with GO Terms
GO:0072643	IFN-γ secretion	483
GO:0061702	inflammasome complex	463
GO:0004998	transferrin receptor activity	344
GO:0042116	macrophage activation	897
GO:0002224	toll-like receptor signalling pathway	1371
Total number in all GO terms		2388

**Table 2:** Total number of Annotations for GO study of innate immune responses and tick-borne pathogens which might be playing a critical role of NK cell in IFN-γ secretion.

GO ID	GO Terms	Number of Gene Products Associated with GO Terms
GO:0030101	natural killer cell activation	122
GO:1902713	regulation of interferon-gamma secretion	35
GO:0072643	interferon-gamma secretion	44
GO:0051775	Response to redox state	32
GO:0006568	tryptophan metabolic process	52
GO:0004517	nitric-oxide synthase activity	109
GO:1905076	regulation of interleukin-17 secretion	24
Total number in all GO terms		202

**Table 3:** GO analysis of 2388 annotations; the immune-related terms have been chosen based on FDR *p* < 0.05.

Biological Process	No. of Genes	Genes Involved	Raw *p*-Value	FDR
regulation of toll-like receptor 3 signaling pathway	9	TIRAP, F2RL1, WDFY1, FLOT1, PELI1, CAV1, TNFAIP3, Ptpn22, UBQLN1	6.14 × 10^−05^	7.16 × 10^−04^
regulation of interferon-gamma secretion	12	Nr1h4, HMHB1, Cd160, Cd2, ZC3H12A, Ptpn22, App, Il36rn, CD244, Rasgrp1, ABL1, Lgals9	3.74 × 10^−16^	1.28 × 10^−14^
response to triacyl bacterial lipopeptide	3	Tlr1, TLR2, Cd14	6.14 × 10^−05^	7.15 × 10^−04^
type I interferon production	5×	Trex1, myd88, Irf3, Irf7, TBK1	1.21 × 10^−06^	1.83 × 10^−05^
MyD88-independent toll-like receptor signaling pathway	32	Cd300lf, CD14, TICAM2, PRKCE, BIRC2, TLR3, IKBKB, UBB, TANK, tlr6, Irf3, UBE2D2, CHUK, TRAF3, Irf7, UBC, Tnip3, UBE2D1, CASP8, UBA52, UBE2D3, IKBKE, IKBKG, TLR4, TBK1, RPS27A, FADD, TICAM1, LY96, RIPK1, RAB11FIP2, BIRC3	5.93 × 10^−42^	5.69 × 10^−40^
toll-like receptor 2 signaling pathway	5	TLR2, IRAK1, RIPK2, TNIP2, PIK3AP1	1.60 × 10^−07^	2.73 × 10^−06^
positive regulation of antigen processing and presentation of peptide antigen via MHC class II	2	TREM2, PYCARD	1.27 × 10^−03^	1.14 × 10^−02^
regulation of MyD88-dependent toll-like receptor signaling pathway	4	CD300LF, IRF7, CD300A, IRF1	1.27 × 10^−03^	1.14 × 10^−02^
macrophage apoptotic process	2	IRF3, CTSL	1.27 × 10^−03^	1.14 × 10^−02^
regulation of interleukin-4 biosynthetic process	2	Cd86, IRF-4	1.27 × 10^−03^	1.14 × 10^−02^
macrophage activation involved in immune response	13	TREM2, TREX1, PRKCE, IL33, TYROBP, Syk, DYSF, GRN, SUCNR1, TICAM1, LBP, SBNO2, HAVCR2	1.06 × 10^−17^	3.97 × 10^−16^
regulation of interleukin-18 production	5	TLR9, TLR2, IL10, NLRP9, Cd84	4.91 × 10^−07^	7.86 × 10^−06^
detection of diacyl bacterial lipopeptide	2	TLR6, TLR2	1.27 × 10^−03^	1.12 × 10^−02^
interferon-gamma secretion	9	TCIRG1, VTCN1, LILRB1, ISG15, TRIM27, BTN3A2, GATA3, F2RL1, BTN3A1	1.27 × 10^−12^	3.46 × 10^−11^
interleukin-10 secretion	2	ISG15, F2RL1	1.27 × 10^−03^	1.12 × 10^−02^
regulation of interleukin-1 beta biosynthetic process	6	JAK2, AGER, IFNG, App, TYROBP, AZU1	1.54 × 10^−08^	2.97 × 10^−07^
interleukin-1 beta secretion	7	TLR6, AIM2, CD36, TLR4, NLRC4, F2RL1, TMEM106A	1.45 × 10^−09^	3.09 × 10^−08^
positive regulation of interleukin-18 production	3	TLR9, TLR2, NLRP9	1.06 × 10^−04^	1.19 × 10^−03^
regulation of interleukin-12 production	27	ARRB2, TLR9, AGER, FOXP1, TLR3, IRAK3, IFNG, CD36, LILRB1, TLR2, TRAF6, IL12B, SYK, RIPK2, HSPD1, THBS1, SCIMP, TLR4, RELA, IL10, cd40, HMGB1, IRF1, TLR8, Lgals9, SLAMF1, TIRAP	4.05 × 10^−29^	2.62 × 10^−27^
positive regulation of granzyme B production	2	PTPN22, CD244	2.10 × 10^−03^	1.75 × 10^−02^
regulation of gamma-delta T cell differentiation	2	SYK, PTPRC	3.13 × 10^−03^	2.45 × 10^−02^
iron ion transport	7	TCIRG1, TFR2, TFRC, ATP6V1G2, LTF, TF, SLC11A1	1.53 × 10^−04^	1.66 × 10^−03^
regulation of inflammatory response to wounding	2	AGER, Grn	3.13 × 10^−03^	2.43 × 10^−02^
positive regulation of type I interferon-mediated signaling pathway	6	IRF3, IRF7, WNT5A, IKBKE, TBK1, FADD	2.26 × 10^−07^	3.79 × 10^−06^
regulation of epithelial cell apoptotic process	17	ARRB2, JAK2, IL6, Il4, CD160, PDPK1, FGB, IL13, Tnf, TNFAIP3, FGA, THBS1, cd40, GATA3, TNIP2, FGG, ABL1	6.20 × 10^−14^	1.88 × 10^−12^
negative regulation of inflammatory response to antigenic stimulus	4	FCGR2B, IL12B, NLRP6, IL10	9.48 × 10^−05^	1.07 × 10^−03^
negative regulation of interferon-beta production	4	PYCARD, LILRB1, CACTIN, PTPRS	1.20 × 10^−04^	1.33 × 10^−03^
cellular response to interferon-beta	6	TREX1, TLR3, AIM2, IFNB, IRF1, ACOD1	2.80 × 10^−06^	4.02 × 10^−05^
interleukin-12-mediated signaling pathway	6	JAK2, IFNG, IL12B, MIF, IL10, IL12A	1.13 × 10^−04^	1.24 × 10^−03^
interferon-gamma-mediated signaling pathway	8	JAK2, IFNGR1, IRF4, IFNG, IRF3, Ifngr2, b2m, IRF1	3.02 × 10^−06^	4.30 × 10^−05^
macrophage activation involved in immune response	50	Csf2, SNCA, JAK2, AGER, TREM2, TREX1, ITGB2, PRKCE, FOXP1, TLR3, C1QA, TRPV1, MAPT, IFNGR1, JUN, IL33, ITGAM, IFNG, TLR6, CX3CL1, TLR2, TLR7, CRTC3, CLU, IL13, App, SLC7A2, CD93, TYROBP, Tnf, SYK, DYSF, EDN2, FPR2, SUCNR1, AIF1, JMJD6, GRN, NAMPT, TLR4, AZU1, TICAM1, Ifngr2, TLR1, LBP, TLR8, TMEM106A, SLC11A1, SBNO2, HAVCR2, C5AR1	1.06 × 10^−17^	3.97 × 10^−16^
regulation of macrophage cytokine production	7	Irak3, CD36, LILRB1, WNT5A, CD74, TLR4, RTN4	6.92 × 10^−08^	1.23 × 10^−06^
macrophage chemotaxis	5	CX3CL1, SFTPD, AZU1, EDN2, CCL3	6.62 × 10^−06^	8.87 × 10^−05^
negative regulation of macrophage apoptotic process	2	CLU, LDLR	5.72 × 10^−03^	4.13 × 10^−02^
Total genes	166			
immune system process	28			
regulation of immune system process	18			
